# Vitamin D Level and Risk of Community-Acquired Pneumonia and Sepsis

**DOI:** 10.3390/nu6062196

**Published:** 2014-06-10

**Authors:** Anna J. Jovanovich, Adit A. Ginde, John Holmen, Kristen Jablonski, Rebecca L. Allyn, Jessica Kendrick, Michel Chonchol

**Affiliations:** 1 Division of Renal Diseases and Hypertension, University of Colorado Denver, Denver, CO 80045 USA; E-Mails: Anna.Jovanovich@ucdenver.edu (A.J.J.); Kristen.Nowak@ucdenver.edu (K.J.); Jessica.Kendrick@ucdenver.edu (J.K.); 2 Department of Emergency Medicine, University of Colorado Denver, Denver, CO 80045 USA; E-Mail: Adit.Ginde@ucdenver.edu; 3 Intermountain Healthcare, Salt Lake City, UT 84157, USA; E-Mail: John.Holmen@imail.org; 4 Denver Health Medical Center, Denver, CO 80204, USA; E-Mail: Rebecca.Allyn@ucdenver.edu

**Keywords:** vitamin D deficiency, sepsis, community-acquired pneumonia, infection, epidemiology

## Abstract

Previous research has reported reduced serum 25-hydroxyvitamin D (25(OH)D) levels is associated with acute infectious illness. The relationship between vitamin D status, measured prior to acute infectious illness, with risk of community-acquired pneumonia (CAP) and sepsis has not been examined. Community-living individuals hospitalized with CAP or sepsis were age-, sex-, race-, and season-matched with controls. ICD-9 codes identified CAP and sepsis; chest radiograph confirmed CAP. Serum 25(OH)D levels were measured up to 15 months prior to hospitalization. Regression models adjusted for diabetes, renal disease, and peripheral vascular disease evaluated the association of 25(OH)D levels with CAP or sepsis risk. A total of 132 CAP patients and controls were 60 ± 17 years, 71% female, and 86% Caucasian. The 25(OH)D levels <37 nmol/L (adjusted odds ratio (OR) 2.57, 95% CI 1.08–6.08) were strongly associated with increased odds of CAP hospitalization. A total of 422 sepsis patients and controls were 65 ± 14 years, 59% female, and 91% Caucasian. The 25(OH)D levels <37 nmol/L (adjusted OR 1.75, 95% CI 1.11–2.77) were associated with increased odds of sepsis hospitalization. Vitamin D status was inversely associated with risk of CAP and sepsis hospitalization in a community-living adult population. Further clinical trials are needed to evaluate whether vitamin D supplementation can reduce risk of infections, including CAP and sepsis.

## 1. Introduction

Relatively little progress has been made in improving mortality associated with community-acquired pneumonia (CAP) [1] which is a leading cause of death in the United States [2]. Likewise, the incidence of sepsis continues to rise, the population-adjusted incidence of sepsis increased 8.7% per year between 1979 and 2000 [3]. While mortality associated with sepsis has improved [3], it remains a substantial cause of death in the United States [2].

The major circulating form of vitamin D, 25-hydroxyvitamin D (25(OH)D), and its active form, 1,25-dihydroxyvitamin D (1,25(OH)_2_D), were originally recognized as important endocrine hormones in calcium homeostasis and bone health. However, studies over the past twenty years suggest a broader role of 25(OH)D in endothelial function, cell proliferation, and immunity. The vitamin D receptor (VDR) is essentially ubiquitous, including immune cells. It responds to 1,25(OH)_2_D [4,5] and regulates antimicrobial peptides cathelicidin and beta-defensing [5]. Furthermore, 25(OH)D deficiency is common; 32% of the U.S. population have 25(OH)D levels <50 nmol/L and 77% have levels <75 nmol/L [6]. Large epidemiological studies have shown an association between 25(OH)D deficiency and chronic diseases [7] including diabetes [8], renal disease [9], and peripheral vascular disease [10].

Lower serum 25(OH)D levels are associated with increased risk of upper respiratory tract infection [11,12,13]. When measured during hospital admission for acute illness, 25(OH)D deficiency is associated with increased risk of mortality in patients with CAP [14], more severe sepsis [15], and mortality in septic patients [16]. Most studies linking infection risk with 25(OH)D levels measure 25(OH)D during acute illness, which may not reflect pre-illness 25(OH)D status. There are no data assessing existing 25(OH)D deficiency with risk of hospital admission for CAP or sepsis. In this case-control study of community-living adults, serum 25(OH)D levels and the risk of hospital admission for CAP and sepsis was evaluated.

## 2. Experimental Section

### 2.1. Data Source

A retrospective matched cohort study was performed using the Intermountain Healthcare Enterprise Data Warehouse, which incorporates comprehensive electronic health and administrative data for over 10 years [17]. Intermountain Healthcare is a non-profit organization with 22 hospitals and over 150 outpatient clinics that serves the states of Utah and southeastern Idaho. The latitude of the hospitals and outpatient clinics is approximately 40° N. Facilities range from major adult tertiary-level care centers to small clinics and hospitals that are the only source of care in rural communities. There were 160,979 admissions from 2008 to 2010 [17]. The institutional review board at Intermountain Healthcare System and University of Colorado Denver approved the project.

### 2.2. Cohort Definition

Case and control selection occurred between 1 January 2008 and 31 December 2010. CAP cases were identified through ICD-9 codes (480–488) and confirmed with chest radiograph. There were 187,132 CAP admissions of which 43,460 had discharge ICD-9 codes indicating pneumonia. Of these, 11,455 had chest radiography confirming pneumonia. 4352 Sepsis cases were identified through ICD-9 codes (995.91, 995.92). Controls were randomly selected from a pool of 62,757 adult patients without a CAP or sepsis diagnosis admitted within the same time period and matched 1:1 with cases by age, sex, race, and season of 25(OH)D measurement. Cases and control had to have a serum 25(OH) level in the electronic medical record 3–15 months prior to admission; therefore, only 132 and 422 patients were included in the final CAP and sepsis analyses, respectively. These time points were chosen arbitrarily to assess the relationship between pre-infection serum 25(OH)D levels and infectious episodes.

### 2.3. 25(OH) D Measurements

Serum 25(OH)D level were measured in all participants using an INCSTAR 25(OH)D two step assay procedure with a coefficient of variation of less than 10%. The first step in the procedure involves the rapid extraction of 25(OH)D from the serum using acetonitrile. Following extraction, the treated sample is assayed by using an equilibrium radioimmunoassay procedure. This method is based on an antibody with specificity to 25(OH)D. The sample, antibody, and tracer are incubated at 20–25 °C for ninety minutes. A second antibody-precipitating complex is used to achieve phase separation. The radioimmunoassay method tends to overestimate the level of 25(OH)D because the antibody recognizes all forms of dihydroxy-vitamin D and D steroids.

### 2.4. Statistical Analysis

The associations of serum 25(OH)D levels with CAP or sepsis admission were evaluated separately. The χ^2^ Test of Independence tested the distribution of categorical variables and the Wilcoxon Rank Sum tested for differences in 25(OH)D levels among cases and controls. Cox logistic regression was performed using log-transformed 25(OH)D as a continuous variable and non-transformed 25(OH)D as a categorical variable (<75 nmol/L *vs*. ≥75 nmol/L, <50 nmol/L *vs*. ≥50 nmol/L, and <37 nmol/L *vs*. ≥37 nmol/L). These thresholds were chosen using established definitions of 25(OH)D deficiency/insufficiency [18,19]. Models adjusted for diabetes, renal disease, and peripheral vascular disease, which were chosen as confounding variables on the basis of previous studies [20,21] and obtained from the Charlson Comorbidity Index score. Two-tailed values of *p* < 0.05 were considered statistically significant.

## 3. Results

### 3.1. Community-Acquired Pneumonia

The demographic and clinical characteristics of the 66 cases and 66 controls for the CAP cohort are described in Table 1. The mean (SD) age of the participants was 60 ± 17 years, 71% were female, and 86% were Caucasian. There was no statistically significant difference in median [IQR] 25(OH)D levels in controls *vs*. cases (79.3 [71.1–88.1] *vs*. 70.1 [62.2–79.6] nmol/L, *p* = 0.33). Renal disease was more prevalent in cases than controls (31.8% *vs*. 10.6%, *p* = 0.003). Serum 25(OH)D levels were recorded in 28 cases 3–5 months prior, in 18 cases 6–11 months prior, in 10 cases each 9–11 and 12–15 months prior to admission.

**Table 1:** Baseline characteristics for community-acquired pneumonia and sepsis cases and matched controls.

	Case	Control
**Number**		
CAP	66	66
Sepsis	211	211
**Age in years**		
CAP	60 ± 17	60 ± 17
Sepsis	65 ± 14	65 ± 14
**Females**		
CAP	47	47
Sepsis	125	125
**Race—White**		
CAP	57	57
Sepsis	189	189
**Race—Hispanic**		
CAP	6	6
Sepsis	13	13
**Race—Other**		
CAP	3	3
Sepsis	5	5
**Diabetes**		
CAP	24 (36.4%)	19 (28.8%)
Sepsis *	97 (46.0%)	64 (30.3%)
**Renal Disease**		
CAP *	21 (31.8%)	7 (10.6%)
Sepsis *	76 (36.0%)	46 (21.8%)
**Peripheral Vascular**		
CAP	13 (19.7%)	9 (13.6%)
Sepsis	61 (28.9%)	45 (21.3%)
25(OH)D (nmol/L) **		
CAP	70.1 [62.2–79.6]	79.3 [71.1–88.1]
Sepsis	61.2 [55.9–66.4]	69.1 [64.2–74.1]
**25(OH)D >75 nmol/L**		
CAP	34 (52.3%)	35 (53.0%)
Sepsis	84 (39.8%)	99 (46.9%)
**25(OH)D 51–75 nmol/L**		
CAP	19 (28.8%)	19 (28.8%)
Sepsis	56 (26.5%)	60 (28.4%)
**25(OH)D 37–50 nmol/L**		
CAP	7 (10.6%)	10 (15.2%)
Sepsis	31 (14.7%)	29 (13.7%)
**25(OH)D <37 nmol/L**		
CAP	9 (9.1%)	2 (3.0%)
Sepsis *	40 (19.0%)	23 (10.9%)

* *p* < 0.05; ** median [IQR]; CAP, community-acquired pneumonia; 25(OH)D, 25 hydroxyvitamin D.

In unadjusted logistic regression, log-transformed 25(OH)D as a continuous variable was not associated with CAP (0.99 [0.90–1.09], *p* = 0.84). A lack of association remained after adjustment for diabetes, renal disease, and peripheral vascular disease (OR 0.94 [0.59–1.48], *p* = 0.78). Likewise, 25(OH)D <75 nmol/L *vs*. ≥75 nmol/L and <50 nmol/L *vs*. ≥50 nmol/L were not associated with CAP in adjusted analyses (Table 2). However, when 25(OH)D was categorized as <37 nmol/L *vs*. ≥37 nmol/L, there was an association with increased odds of CAP (OR 2.57 [1.08–6.08], *p* = 0.03; Table 2) in the adjusted model.

**Table 2:** Adjusted odds ratios for Community-Acquired Pneumonia (CAP) and Sepsis Cases relative to controls by serum 25(OH)D levels.

Model ^§^	OR (95% CI)	*p*-Value
**25(OH)D <75 nmol/L *vs*. ≥75 nmol/L**		
CAP	1.03 (0.51–2.09)	0.93
Sepsis	1.24 (0.84–1.83)	0.28
**25(OH)D <50 nmol/L *vs*. ≥50 nmol/L**		
CAP	0.96 (0.35–2.61)	0.94
Sepsis	1.75 (1.11–2.77)	0.02
**25(OH)D <37 nmol/L *vs*. ≥37 nmol/L**		
CAP	2.57 (1.08–6.08)	0.03
Sepsis	1.89 (1.09–3.31)	0.02

**^§ ^**Adjustments made for diabetes, renal disease, peripheral vascular disease; CAP, community-acquired pneumonia; 25(OH)D, 25 hydroxyvitamin D.

### 3.2. Sepsis

The demographic and clinical characteristics of the 211 cases and 211 controls for the sepsis cohort are described in Table 1. The mean (SD) age of the participants was 65 ± 14 years, 59% were female and 91% were Caucasian. There was no statistically significant difference in median [IQR] 25(OH)D levels in controls *vs*. cases (69.1 [64.2–74.1] nmol/L *vs*. 61.2 [55.9–66.4] nmol/L, *p* = 0.05). Comorbid conditions were more prevalent in sepsis cases than in controls: diabetes (46.0% *vs*. 30.3%, *p* = 0.0009) and renal disease (36.0% *vs*. 21.2%, *p* = 0.001). Serum 25(OH)D levels were recorded in 93 cases 3–5 months prior, in 50 cases 6–11 months prior, in 47 cases 9–11 months prior, and 21 cases 12–15 months prior to admission.

In unadjusted logistic regression, log-transformed 25(OH)D as a continuous variable was not associated with sepsis (OR 0.99 [0.93–1.05], *p* = 0.70). A lack of association remained after adjustment for diabetes, renal disease, and peripheral vascular disease (OR 0.82 [0.64–1.05], *p* = 0.12). 25(OH)D <75 nmol/L *vs*. ≥75 nmol/L was not associated with sepsis in adjusted analyses (Table 2). However, when 25(OH)D was categorized as <50 nmol/L *vs*. ≥50 nmol/L and <37 nmol/L *vs*. ≥37 nmol/L, there was an association with increased odds of sepsis (Table 2, OR 1.75 [1.11–2.77], *p* = 0.02 and 1.89 [1.09–3.31], *p* = 0.02, respectively) in adjusted analyses.

## 4. Discussion

In a cohort of community-living adults, increased risk of hospitalization for CAP was associated with serum 25(OH)D levels <37 nmol/L and for sepsis with serum 25(OH)D levels <50 nmol/L. This association was not observed for 25(OH)D <75 nmol/L, suggesting that 25(OH)D <37 nmol/L confers a greater risk of infection than vitamin D insufficiency.

These findings are consistent with other epidemiologic studies linking vitamin D deficiency with increased risk of infection and infection-associated complications. A large observational study using National Health and Nutrition Examination Survey data showed that in a diverse cohort of 18,883 individuals greater than 12 years of age, those with a serum 25(OH)D level <25 nmol/L and a serum 25(OH)D 25–75 nmol/L had a 36% and 24% increased risk of upper respiratory tract infection, respectively, compared to those with a serum 25(OH)D ≥75 nmol/L [11]. Likewise, in a prospective observational study evaluating 25(OH)D levels in Finnish military recruits, 25(OH)D levels <40 nmol/L were associated with a higher likelihood of physician diagnosed respiratory tract infections and lost days of work in the subsequent six months [13]. Ginde and colleagues reported that upon presentation to an urban emergency department, 81 patients with sepsis and 25(OH)D levels <75 nmol/L were more likely to have severe sepsis compared to those with 25(OH)D levels ≥75 nmol/L (61% *vs*. 24%, *p* = 0.006) at initial evaluation and at 24 h (67% *vs*. 29%, *p* = 0.005) [15]. While other studies have measured 25(OH)D levels at acute illness onset, our study evaluates 25(OH)D levels at least 3 months prior to hospital admission, thus suggesting that there is an increased risk of CAP with a 25(OH)D level <37 nmol/L and sepsis with a 25(OH)D level <50 nmol/L. It also eliminates the potential for confounding by acute illness altering serum 25(OH)D levels.

Both the VDR and CYP27B1, the gene encoding 1-α-hydroxylase, which converts 25(OH)D to its active form 1,25(OH)_2_D, are expressed in immune cells, suggesting that 25(OH)D has paracrine or autocrine function. Furthermore, 1-α-hydroxylase in macrophages is not regulated by parathyroid hormone (PTH) [22] but depends on circulating 25(OH)D concentrations or may be induced by cytokines [23]. When toll-like receptors on macrophages bind bacterial wall lipopolysaccharides (LPS), 1-α-hydroxylase and VDR expression is increased^4^ resulting in local conversion of 25(OH)D to 1,25(OH)_2_D, which in turn increases the expression of cathelicidin and beta-defensing, bactericidal proteins. There is evidence that cathelicidin transcription is particularly dependent on sufficient levels of 25(OH)D [4]. Indeed, in a study of critically ill patients with and without sepsis, Jeng and colleagues report a positive relationship between 25(OH)D levels and LL-37 (cathelicidin) levels [24].

In sepsis, 25(OH)D and 1,25(OH)_2_D may have other effects beyond those associated with immunity such as endothelial function, coagulation, and hemodynamic stability. In rats with induced sepsis, pretreatment with 1,25(OH)_2_D resulted in a more normal coagulation profile compared to placebo [25]. The VDR is also found in arterioles and the myocardium [26] and 1,25(OH)_2_D has been shown to enhance the effect of inotropes [27] suggesting a possible positive hemodynamic effect of 1,25(OH)_2_D in sepsis [25]. Taken together, sufficient circulating 25(OH)D levels, independent of the classic vitamin D-PTH axis, play a pivotal role in immunity. Moreover, 25(OH)D may also be protective against the adverse physiologic changes that occur in sepsis.

The findings of this study are consistent with other epidemiologic reports linking serum 25(OH)D <75 nmol/L with increased risk of infection and associated complications [11,12,13,14,15,16]. While other studies measured 25(OH)D levels during acute illness, this is the first to evaluate 25(OH)D levels ≥3 months prior to hospital admission for CAP or sepsis, eliminating potential confounding by acute illness altering serum 25(OH)D levels. This study has important strengths. Chest radiograph confirmed CAP ICD-9 codes. Serum 25(OH)D level was measured prior to the onset of illness, which is different from other studies that measured 25(OH)D at the time of presentation. This is important because vitamin D binding protein may be decreased resulting in potential urinary wasting of 25(OH)D in sepsis [24]. Finally, this study used a community-living cohort that included a relatively large number of patients with sepsis.

This study has several limitations. First, it is retrospective and observational so causation cannot be assumed. Second, the use of ICD-9 codes to identify admissions for sepsis limits details of cause and severity. Third, while cases and controls were well-matched and regression models adjusted for comorbidities, there may be other unmeasured confounders that could potentially affect the results. Fourth, ascertainment bias may exist because only cases and controls with a serum 25(OH)D level available prior to admission were considered. Additionally, no information is available on outcomes during the hospitalization.

## 5. Conclusions

In conclusion, serum 25(OH)D level <37 nmol/L in a community-living cohort was associated with increased risk of hospital admission for CAP and sepsis. Large randomized controlled trials are needed to establish whether or not 25(OH)D repletion will decrease CAP and sepsis incidence in community-living adult populations.
